# Recent advances in understanding neurotrophin signaling

**DOI:** 10.12688/f1000research.8434.1

**Published:** 2016-07-28

**Authors:** Mark Bothwell

**Affiliations:** 1 Department of Physiology & Biophysics, University of Washington, Seattle, WA, USA

**Keywords:** 75 kDa neurotrophin receptor, proneurotrophins, mature processed neurotrophins, Spätzle proteins

## Abstract

The nerve growth factor family of growth factors, collectively known as neurotrophins, are evolutionarily ancient regulators with an enormous range of biological functions. Reflecting this long history and functional diversity, mechanisms for cellular responses to neurotrophins are exceptionally complex. Neurotrophins signal through p75 ^NTR^, a member of the TNF receptor superfamily member, and through receptor tyrosine kinases (TrkA, TrkB, TrkC), often with opposite functional outcomes. The two classes of receptors are activated preferentially by proneurotrophins and mature processed neurotrophins, respectively. However, both receptor classes also possess neurotrophin-independent signaling functions. Signaling functions of p75 ^NTR^ and Trk receptors are each influenced by the other class of receptors. This review focuses on the mechanisms responsible for the functional interplay between the two neurotrophin receptor signaling systems.

Nerve growth factor (NGF) and its orthologs are collectively known as neurotrophins. Mammals have four neurotrophins – NGF, brain-derived neurotrophic factor (BDNF), neurotrophin-3 (NT-3) and neurotrophin 4 (NT-4, also known as NT-4/5). Neurotrophins, functioning as homodimers, have a wide variety of functions in both neural and non-neural tissues, and they control adult physiology as well as embryonic development.

The 4 neurotrophins signal through three paralogous receptor tyrosine kinases (TrkA, TrkB and TrkC) and the 75 kDa neurotrophin receptor (p75 ^NTR^), which is a member of the death domain-containing receptor subgroup (so-called death receptors) of the TNF receptor superfamily. While p75 ^NTR^ is activated by all four neurotrophins, the Trk receptors are more selective, as shown in the table.

**Table 1.** Ligand preferences of neurotrophin receptors. Ligands listed in italic type have lower affinity and/or are less commonly important for receptor activation *in vivo*.

Receptor	Ligand
p75 ^NTR^	NGF, BDNF, NT3, NT4
TrkA	NGF, *NT3*
TrkB	BDNF, NT4, *NT3*
TrkC	NT3

The neurotrophin system is ancient, as orthologs of neurotrophins, p75 ^NTR^ and Trks are found in invertebrates as diverse as sea urchins, mollusks and round worms ^1, 2^. Consequently, this signaling system has had half a billion years of evolution to develop extraordinary complexity. A goal of this review will be to capture the many levels of complexity of the neurotrophin receptor signaling system. Perversely, the entire signaling system has been lost in Caenorhabditis and Drosophila lineages, depriving investigators of convenient genetic systems to unravel the complexity of neurotrophin signaling.

Although neurotrophic Drosophila proteins have been referred to as neurotrophins, they are only distantly similar to neurotrophins of other invertebrate and vertebrate species, and they signal via toll-like receptors, rather than p75 ^NTR^ or Trk-like receptors ^3– 5^. I prefer to refer to these neurotrophin-like cytokines by their original names, Spätzle and Spätzle-family proteins, rather than as neurotrophins, to avoid confusion.

It has been said that there is a yin and yang relationship between p75 ^NTR^ and Trk receptors, because they often are co-expressed and function oppositely ^6^. For example, TrkA signaling in sympathetic neurons promotes axon growth and neuronal survival, whereas p75 ^NTR^ signaling promotes axon degeneration and neuronal cell death ^7^. BDNF controls hippocampal neuronal synaptic plasticity, learning and memory with TrkB signaling promotes synaptic long term potentiation (LTP) and p75 ^NTR^ signaling promoting long term depression (LTD) ^6^. Functional interactions between the two receptor systems produce multiple levels of complexity, while several different mechanisms control the balance between the yin and the yang of neurotrophin signaling.

Like many biologically active polypeptides, neurotrophins are synthesized as precursors (pro-neurotrophins), which are cleaved to release an N-terminal prodomain peptide and a C-terminal mature neurotrophin. This cleavage event may occur either within the secretory pathway or following secretion, so that receptors may be exposed to both proneurotrophins and mature neurotrophins. Importantly, p75 ^NTR^ binds both mature and proneurotrophins, and is more effectively activated by proneurotrophins, while only mature neurotrophins activate Trk receptors ^8, 9^. The enhanced action of proneurotrophins binding to p75 ^NTR^ is dependent on association of p75 ^NTR^ with sortilin or SorCS2, Vps10p-domain proteins which bind a conserved motif in proneurotrophin prodomains ^10, 11^.

The complexity of function that can be generated by these relationships is well illustrated by sympathetic neurons, which express TrkA and p75 ^NTR^, but not TrkB. For these neurons, proNGF, which activates p75 ^NTR^ but not TrkA, promotes cell death. Mature NGF, which activates both p75 ^NTR^ and TrkA, promotes cell survival. ProBDNF or mature BDNF promotes cell death, because these ligands bind p75 ^NTR^ but not TrkA ^12– 14^.

The canonical mode of signaling by Trk receptors is similar to signaling by other receptor tyrosine kinases. Neurotrophin binding promotes formation of Trk dimers, and induces trans-phosphorylation of Trk cytoplasmic domain tyrosine residues, initiating recruitment of signaling adapter proteins that foster signaling by ras/ERK1/2, PI3 kinase/Akt STAT and phospholipase Cγ pathways ^15^. However, alternatively spliced forms of TrkB and TrkC (misleadingly known as truncated TrkB and truncated TrkC) lack a tyrosine kinase domain, but possess alternative cytoplasmic domain sequences that signal by less extensively characterized mechanisms ^16, 17^.

One feature of canonical signaling by Trk receptors that differs from many other receptor tyrosine kinases is the use of so-called signaling endosomes to achieve retrograde axonal signaling. For most receptor tyrosine kinases, ligand-mediated activation of the receptor leads to receptor endocytosis, followed either by lysosomal degradation of the receptor or recycling back to the cell surface. However, in many physiological scenarios, the survival and/or differentiated state of neurons is regulated by neurotrophins secreted by the target tissues those neurons innervate. In this context, endocytosis of the neurotrophin/Trk complex generates signaling endosomes, which undergo retrograde axonal transport, delivering the activated neurotrophin/receptor complex to the somatic compartment in order to permit control of nuclear transactivation of genes ^18– 21^. The exquisite complexity associated with this mode of signaling is nicely illustrated by sympathetic neurons, where NT3 and NGF differently control axonal TrkA signaling functions because of differences in the pH-dependence for NT3 and NGF binding. Sympathetic axons encounter NT3 on the route to their target. NT3 activates TrkA and achieves local control of axonal growth cone dynamics, but does not engage signaling to the cell soma because NT3 dissociates from TrkA at the acidic pH within endosomes, causing TrkA receptors to recycle to the local plasma membrane without production of axonally transported signaling endosomes. In contrast, NGF/TrkA complexes remain intact as endosomes acidify, allowing TrkA to engage the motor systems that mediate retrograde axonal transport of TrkA-bearing endosomes ^22^.

p75 ^NTR^ signaling shares several features with other death receptors. A juxta-membrane region of the cytoplasmic domain binds TRAF6, which engages signaling pathways leading to activation of NF-κB and JNK ^23, 24^. The death domain interacts with RhoGDI, which controls RhoA activation, and RIP2 kinase, which contributes to NF-κB and JNK activation ^25^. Neurotrophin binding to p75 ^NTR^ inhibits RhoA activation ^26^, while enhancing JNK activation ^27, 28^. However, TRAF6, RhoGDI and RIP2 are only a few of the bewildering array of p75 ^NTR^-binding signaling adapter proteins that have been reported to mediate p75 ^NTR^ signaling. Other notable examples include NRIF, which promotes JNK activation ^28, 29^, MAGE proteins including NRAGE, which promote Rac1 and JNK activation ^30^, and Bex1, which negatively affects NF-κB signaling ^31, 32^. Further, p75 ^NTR^ has been reported to influence glucose uptake in adipocytes via Glut4 by directly binding the trafficking regulators Rab5 and Rab31 ^33^, to controls energy expenditure in obese mice on a high-fat diet by inhibiting cAMP signaling in adipocytes via direct association of p75 ^NTR^ with protein kinase A ^34^, and to promote fibrinolysis in nerve injury and lung fibrosis by binding and enhancing the cAMP degradative activity of phosphodiesterase PDE4A4/5 ^35^.

One feature of p75 ^NTR^ function is unique, so far, among known receptors. A cysteinyl residue in the membrane spanning domain of p75 ^NTR^ forms a disulfide bond, within the lipid bilayer, creating a covalently linked-dimeric form of p75 ^NTR^, and this covalent linkage is required for neurotrophin-dependent JNK activation, but not inhibition of RhoA activity ^36^. Mutation of the single cysteinyl residue required to form this disulfide bond eliminates p75 ^NTR^-dependent death signaling in neurons *in vitro* and *in vivo* ^37^. A physiologically occurring disulfide bond within a lipid bilayer has never been described previously in any membrane protein, and the mechanism by which a disulfide forms in such an unusual environment is unclear. However, our unpublished evidence (Leslayann Schecterson and Mark Bothwell) demonstrates that this linkage forms within 2 minutes when cells are exposed to minute concentrations of hydrogen peroxide, indicating that oxidative stress may control p75 ^NTR^ signaling, as we have reported previously ^38^.

A detailed model, illustrated in Figure 1, has recently been proposed for death domain-mediated p75 ^NTR^ signaling ^25^. In the absence of bound neurotrophin, the death domains of p75 ^NTR^ dimers form a homodimeric complex. The RhoGDI binding site is not occluded by this interaction, so non-liganded p75 ^NTR^ engages RhoGDI-dependent RhoA activation. Binding of a neurotrophin dimer to the extracellular domain of a disulfide-linked p75 ^NTR^ dimer, causes a scissoring action (or more accurately a snail-tong action) around the disulfide pivot-point, separating originally juxtaposed death domains, and allowing access of RIP2 to a binding site that was previously partially occluded by the death domain/death domain interaction. The RIP2 binding site partially overlaps with the RhoGDI binding site, so RIP2 binding displaces RhoGDI, initiating JNK activation and terminating RhoA activation.

**Figure 1.**
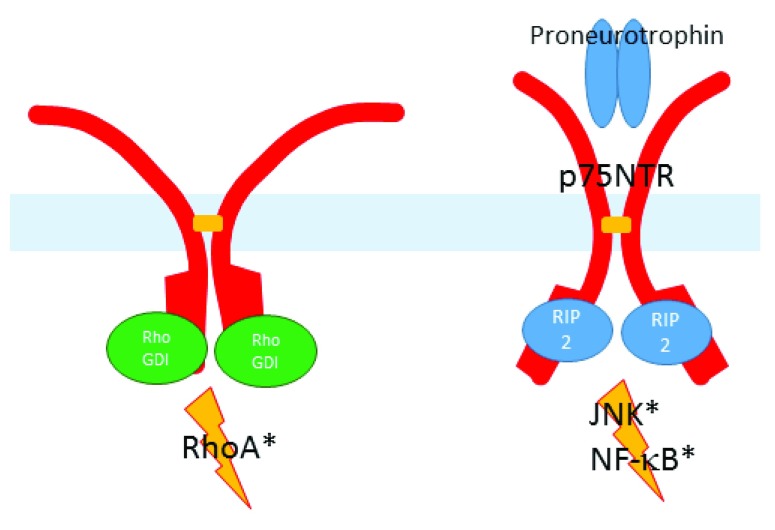
p75 ^NTR^ Signaling. In absence of ligand, death domains of disulfide-linked p75 ^NTR^ dimer bind RhoGDI, promoting formation of active GTP-bound RhoA. Binding of neurotrophin or proneurotrophin causes a scissoring action of the dimer, displacing the death domains laterally and allowing RIP2 to bind the death domains. RIP2 promotes activation of JNK and NF-κB and by displacing RhoGDI, terminates RhoA activation.

Challenging the elegant simplicity of this model, an element of controversy has been introduced by the suggestion that the p75 ^NTR^ oligomer observed on non-reducing SDS gels is a trimer, rather than a dimer ^39, 40^. This conclusion relies primarily on the ratio of the apparent molecular weights of p75 ^NTR^ monomer and oligomer on non-reducing SDS gels. A caveat for such analysis is that the theoretical basis for the proportionality of electrophoretic mobility and protein mass on SDS gels assumes that SDS-induced denaturation fully unfolds the protein and causes the protein to form linear structures with length proportional to mass ^41^. This assumption does not apply to p75 ^NTR^, which contains multiple intra-chain disulfide linkages if disulfide bonds are not reduced before electrophoresis. p75 ^NTR^ function as a trimer is inconsistent with X-ray crystallographic and/or NMR generated three-dimensional structures indicating that the extracellular domain of p75 ^NTR^ ^42^, the death domain region of the intracellular domain of p75 ^NTR^ ^25^, and the membrane spanning domain of p75 ^NTR^ ^43^ each forms dimers, not trimers. Application of emerging technologies such as cryo-EM will be required to provide definitive evidence about the stoichiometry of intact p75 ^NTR^.

Although the reader may think that the preceding account is already quite complicated enough, another mode of p75 ^NTR^ signaling, and its manner of influence by Trk receptors, provides substantial additional complexity, as summarized in Figure 2. Soon after the discovery of Trk receptors, it was reported that p75 ^NTR^/Trk heterodimeric complexes could form, enhancing the affinity of NGF binding to TrkA ^44^, and causing TrkA and TrkB to be less effectively activated by NT3 ^15^. Although the physiological importance of these p75 ^NTR^ effects on Trk signaling remain uncertain, recently Trk-dependent effects on p75 ^NTR^ signaling have emerged that seem likely to have physiological relevance. p75 ^NTR^ has an alternative signaling pathway that resembles the mode of signaling of Notch. ADAM10 or ADAM17-dependent cleavage of the p75 ^NTR^ extracellular domain near the membrane, followed by γ-secretase mediated release of the intracellular domain into the cytoplasm, fosters signaling ^45– 47^. Interestingly, a similar mode of signaling by another TNF receptor superfamily member, TNFR1, has been described recently ^48^. Differential cleavage of p75 ^NTR^ in different types of neurons produced different signaling outcomes ^49^. Signaling effects attributed to the mobilized p75 ^NTR^ intracellular domain include nuclear accumulation of NRIF ^47^, association with the ubiquitin ligase siah2 controlling degradation of the transcription factor Hif1α ^50^, and association with nuclear pore complexes promoting nuclear uptake of the SMAD2 transcription factor ^51^. Neurotrophin binding to p75 ^NTR^ does not directly influence the rate of ADAM protease-mediated cleavage of p75 ^NTR^ ^45, 46, 52^ although signaling pathways initiated by neurotrophin binding may enhance p75 ^NTR^ cleavage many hours after neurotrophin exposure ^47^. Interestingly, activation of Trk receptors promotes the p75 ^NTR^ cleavage pathway ^45, 53^.

**Figure 2.**
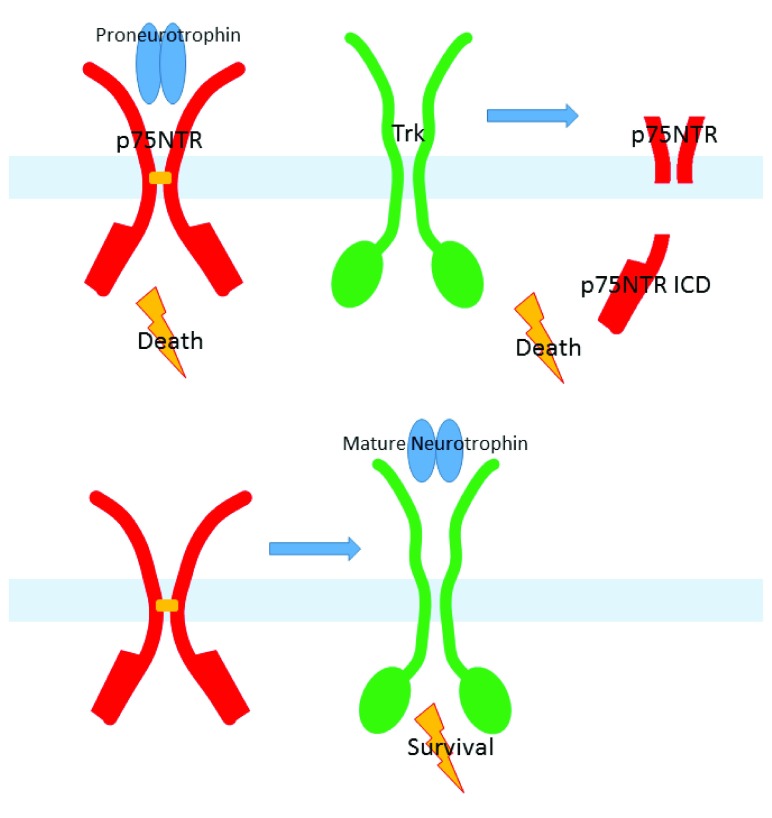
Cell death and cell survival signaling by p75 ^NTR^ and Trk receptors. ( **Above**) Proneurotrophins interact preferentially with p75 ^NTR^, promoting JNK dependent caspase activation and cell death. Sequential cleavage of p75 ^NTR^ by ADAM10/17 and γ-secretase, allowing cytoplasmic mobilization of the intracellular domain of p75 ^NTR^, may also promote cell death, by a mechanism that is only indirectly promoted by neurotrophins. Non-liganded Trk A and TrkC promote cell death by a mechanism that implicates the p75 ^NTR^ cleavage pathway. ( **Below**) Mature neurotrophins preferentially activate Trk receptors, in a manner that may be enhanced by p75 ^NTR^, particularly for TrkA. Neurotrophin activation of Trks promotes cell survival.

It remains to be determined definitively whether Trk activity represents a major mode of regulation of the p75 ^NTR^ cleavage signaling pathways *in vivo*. However, the recently reported function of TrkA and TrkC as dependence receptors may reflect a consequence of this mode of interaction. Dependence receptors are receptors that signal constitutively until ligand binding terminates signaling. Although each of the three Trk paralogs was originally found to promote neuronal survival, in some neuronal populations, neurotrophin-independent effects of TrkA and TrkC were reported to promote neuronal cell death, and the cleavage mediated signaling pathway of p75 ^NTR^ has been implicated as a mediator of this effect ^54, 55^.

The foregoing paragraphs have focused on neurotrophin-dependent signaling by neurotrophin receptors, but other ligands importantly engage signaling by both Trk and p75 ^NTR^ receptors. For Trk receptors, the most common mechanism for signaling in response to non-neurotrophin ligands involves receptor transactivation, most commonly of TrkB. A variety of G protein-coupled receptors, including PACAP and A2a adenosine receptors, activate TrkB via Gsα-dependent activation of Src family kinases (commonly Fyn in neural tissue) ^56, 57^. In the context of embryonic cerebral cortex, where developing neurons express abundant TrkB receptors, EGF-dependent activation of EGF receptors engages Src-dependent TrkB activation ^58^. Src family kinase-mediated Trk transactivation also is induced by ligand-dependent activation of Low-density lipoprotein receptor-related protein 1 (LRP1) ^59^, and by zinc ion, which is co-released during glutamatergic neurotransmission ^60, 61^. One interesting feature of transactivation of TrkB is that activation commonly occurs in the ERGIC or Golgi compartments, rather than at the cell surface. Signaling from intracellular sites may not be functionally equivalent to signaling from the plasma membrane. For example, PACAP-dependent transactivation of TrkB in cultured hippocampal neurons, by coupling to pathways that otherwise control Golgi dynamics during cell division, induces fragmentation of the Golgi apparatus and alters Golgi-dependent processing of other membrane proteins ^62^.

A variety of modes of neurotrophin-independent activation of p75 ^NTR^ have been reported. p75 ^NTR^ is one of several receptors that bind Aβ peptide and putatively engage in pathogenic signaling in Alzheimer’s disease ^40, 63^. Other scenarios in which non-neurotrophin ligands control p75 ^NTR^ signaling involve association of p75 ^NTR^ with co-receptors that bind the activating ligand. Axon--repellant signaling by CNS myelin proteins such as Nogo, MAG, or OMgp, mediated by association of NgR1 with p75 ^NTR^, ^64, 65^ or the p75 ^NTR^ homolog, Troy ^66^ while ephrin-A/p75 ^NTR^ complexes have been implicated as mediators of EPH-dependent reverse signaling ^67^.

Although great progress has been made in elucidating the signaling pathways employed by neurotrophin receptors, a systems level understanding of how these signaling pathways are selectively engaged *in vivo* is sadly lacking. It is a daunting task to understand how this extraordinarily rich palette of neurotrophin receptor signaling modalities is controlled physiologically.

## Abbreviations

BDNF, brain-derived neurotrophic factor; EGF, epidermal growth factor; ERGIC, endoplasmic reticulum Golgi intermediate compartment; JNK, Jun kinase; MAG, myelin associated glycoprotein; NGF, nerve growth factor; NMR, nuclear magnetic resonance; NT3, neurotrophin 3; NT4, neurotrophin 4; OMgp, oligomyelin glycoprotein; p75 ^NTR^, 75 kDa neurotrophin receptor; RhoGDI, Rho GDP dissociation inhibitor; TNF, tumor necrosis factor; Trk, tropomyosin related kinase.
